# Supplementary material for: Single-cell transcriptomic atlas reveals increased regeneration in diseased human inner ear balance organs
Source: Nat Commun. 2024 Jun 6;15:4833. doi: 10.1038/s41467-024-48491-y (PMC11156867; doi:10.1038/s41467-024-48491-y)
Supplement: Supplementary file 1 — Supplementary Information [file 41467_2024_48491_MOESM1_ESM.pdf]

Supplementary Information for

Single-cell transcriptomic atlas reveals increased regeneration in diseased human inner ear balance organs

Tian Wang[1,2], Angela H. Ling[1,3], Sara E. Billings[1], Davood K. Hosseini[1], Yona Vaisbuch[1], Grace S. Kim[1], Patrick J. Atkinson[1], Zahra N. Sayyid[1], Ksenia A. Aaron[1], Dhananjay Wagh[4], Nicole Pham[1], Mirko Scheibinger[1], Ruiqi Zhou[3], Akira Ishiyama[5], Lindsay S. Moore[1], Peter Santa Maria[1], Nikolas H. Blevins[1], Robert K. Jackler[1], Jennifer C. Alyono[1], John Kveton[6], Dhasakumar Navaratnam[6,7], Stefan Heller[1], Ivan A. Lopez[5], Nicolas Grillet[1], Taha A. Jan[3,a], Alan G. Cheng[1,8,a]

[1] Department of Otolaryngology – Head and Neck Surgery, Stanford University School of Medicine, Stanford, CA, 94305, USA

[2] Department of Otolaryngology – Head and Neck Surgery, The Second Xiangya Hospital, Central South University, Changsha, Hunan Province, 410011, PR China.

[3] Department of Otolaryngology – Head and Neck Surgery, Epithelial Biology Center, Vanderbilt University Medical Center, Nashville, TN, 37232, USA

[4] Stanford Genomics Facility, Stanford University School of Medicine, Stanford, CA, 94305, USA

[5] Department of Head and Neck Surgery, University of California Los Angeles, Los Angeles, CA, 90095, USA

[6] Department of Surgery, Yale University School of Medicine, New Haven, CT, 06510, USA

[7] Department of Neurology, Yale University School of Medicine, New Haven, CT, 06510, USA

[8] Lead Contact

[a] Corresponding authors: Taha A. Jan, M.D. (taha.a.jan@vumc.org), Alan G. Cheng, M.D. (aglcheng@stanford.edu).

Figure S1 (to Figure 1) Surgical approaches for procurement and single cell computational quality control for human utricles.

Figure S2 (to Figure 2) Strategies to subset and validate enriched genes in human hair cells and supporting cells.

Figure S3 (to Figure 1) Separate analysis of organ donor and vestibular schwannoma utricles.

Figure S4 (to Figure 3) Genes enriched in hair cell and species comparison.

Figure S5 (to Figure 5) Degenerating hair cells in utricles from vestibular schwannoma patients.

Figure S6 (to Figure 5) Comparative analysis of organ donor and vestibular schwannoma cell types.

Figure S7 (to Figure 6) Trajectory analysis.

Figure S8 (to Figure 7) Characteristics of hair cell precursors in organ donor and vestibular schwannoma utricles.

Figure S9 (to Figure 1) Expression patterns of genes associated with hearing loss and vestibular dysfunction.

Figure S10 (to all figures) gEAR profile demonstrating data accessibility.

Supplementary Data 1: Patient demographic and medical information.

Supplementary figures and legends:

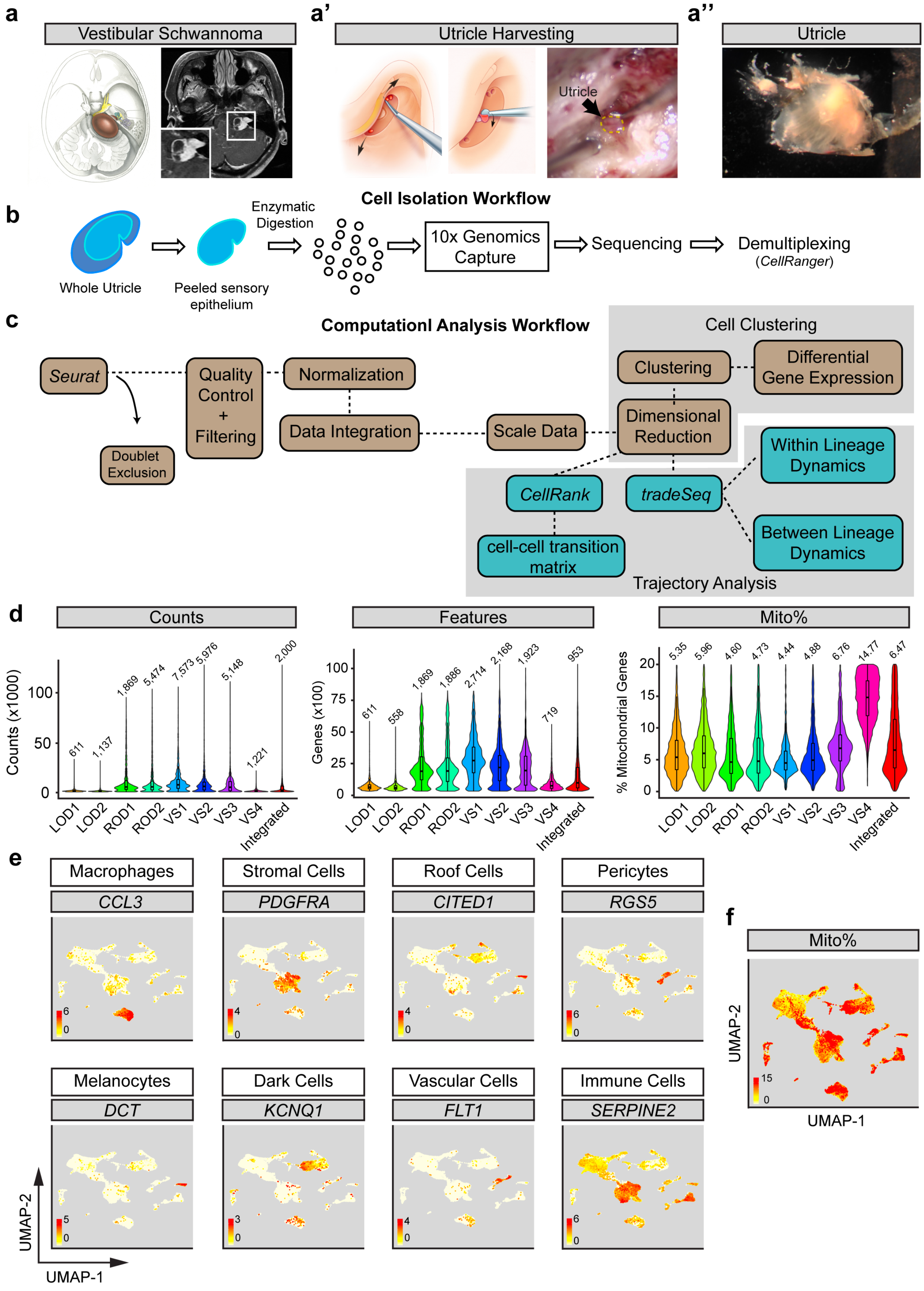

Supplementary Figure 1. Surgical approaches to procure and quality control for human utricles.

(a) Cartoon diagram and axial T1-contrast enhanced MRI showing a vestibular schwannoma (VS) tumor filling the internal auditory canal and compressing the brainstem. (a') Patients undergoing translabyrinthine resection of VS tumors had their utricles harvested from the elliptical recess of the vestibule. (a'') Sample utricle harvested from a VS patient. (b) Whole utricles isolated from VS patients and organ donors (ODs) were microdissected and cleaned under a stereomicroscope, then underwent enzymatic digestion, after which the sensory epithelium was mechanically peeled away. Further digestion and mechanical trituration were used to obtain single cells and cells were submitted for 10X capture. (c) Outline of computational workflow used. *CellRanger* output matrix was imported into *Seurat* where doublet exclusion was first carried out with *DoubletFinder*, followed by quality control and filtering and subsequently outlined steps. (d) Violin plots with boxplot inside showing total read counts, features (genes) and mitochondrial gene percentages per cell. The median number for each category is shown above each plot. LOD1, LOD2, ROD1 and ROD2 are four 10x captures from both left and right utricles from one donor. VS1, VS2, VS3 and VS4 are four 10x captures from different patients. Integrated category shows the post quality control data. (e) Marker genes that met differential expression criteria that were used for cell cluster annotations. Each UMAP plot shows the $\log_2$ expressions for indicated genes with white being 0, yellow as low, and red with maximum expression at indicated levels. (f) UMAP plot with percentage of mitochondrial genes displayed post quality control.

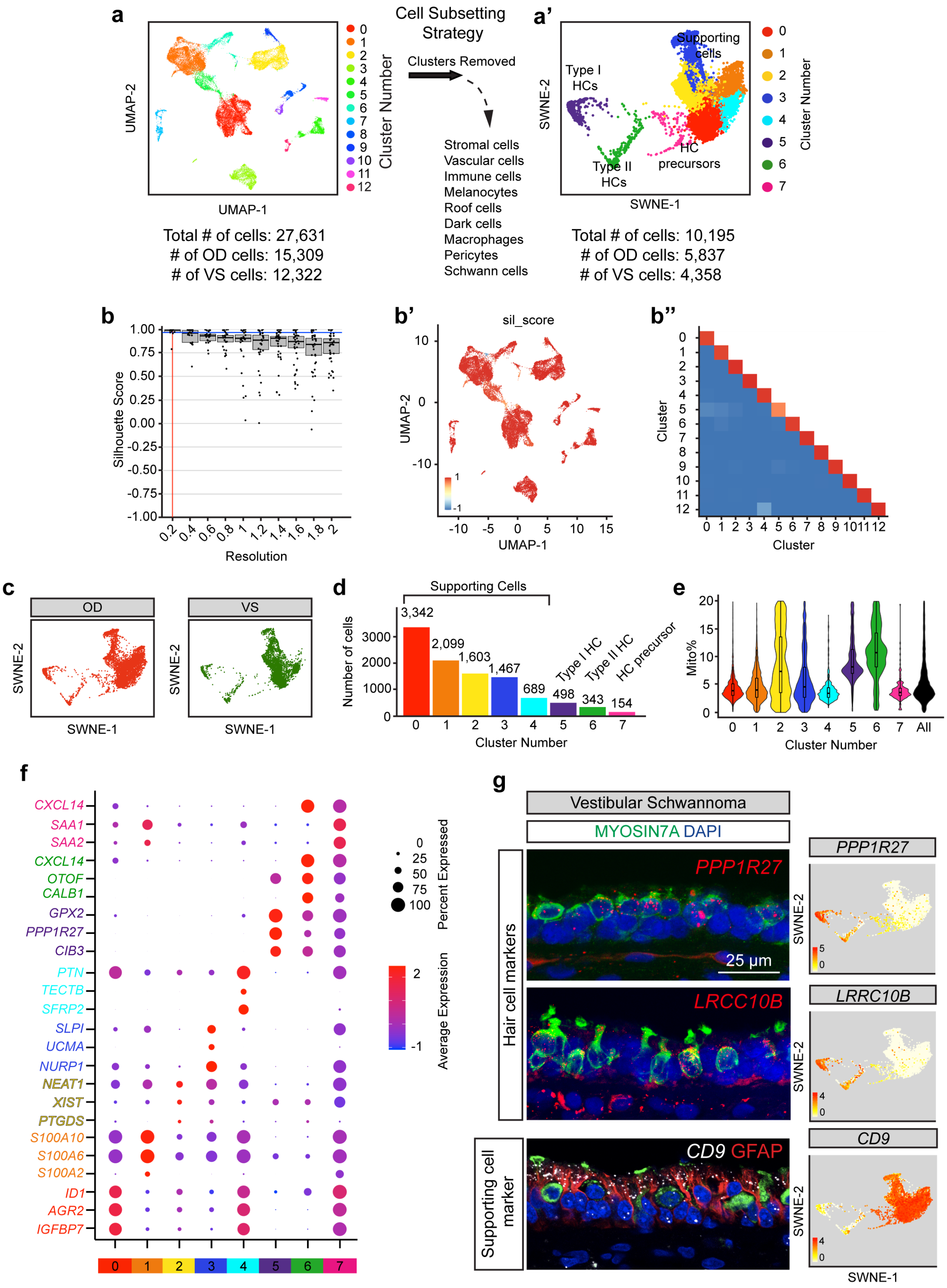
a
UMAP-2
UMAP-1
Cluster Number
0 1 2 3 4 5 6 7 8 9 10 11 12
Total # of cells: 27,631
# of OD cells: 15,309
# of VS cells: 12,322
Cell Subsetting Strategy
Clusters Removed
Stromal cells
Vascular cells
Immune cells
Melanocytes
Roof cells
Dark cells
Macrophages
Pericytes
Schwann cells
a'
SWNE-2
SWNE-1
Supporting cells
Type I HCs
Type II HCs
HC precursors
Cluster Number
0 1 2 3 4 5 6 7
Total # of cells: 10,195
# of OD cells: 5,837
# of VS cells: 4,358
b
Silhouette Score
Resolution
b'
sil_score
UMAP-2
UMAP-1
b''
Cluster
Cluster
c
OD
VS
SWNE-2
SWNE-1
d
Supporting Cells
Number of cells
3,342
2,099
1,603
1,467
689
498
343
154
Type I HC
Type II HC
HC precursor
Cluster Number
e
Mito%
Cluster Number
All
f
CXCL14
SAA1
SAA2
CXCL14
OTOF
CALB1
GPX2
PPP1R27
CIB3
PTN
TECTB
SFRP2
SLPI
UCMA
NURP1
NEAT1
XIST
PTGDS
S100A10
S100A6
S100A2
ID1
AGR2
IGFBP7
Percent Expressed
0 25 50 75 100
Average Expression
g
Vestibular Schwannoma
MYOSIN7A DAPI
Hair cell markers
PPP1R27
25 μm
LRRC10B
Supporting cell marker
CD9 GFAP
PPP1R27
LRRC10B
CD9
SWNE-2
SWNE-1

Supplementary Figure 2. Strategies to subset and validate enriched genes in human hair cells and supporting cells.

(a) UMAP plot integrating cells from organ donor (OD) and vestibular schwannoma (VS) utricles, showing 13 distinct cell clusters as in Fig. 1. Based on known cell type annotations, hair cells, presumed hair cell precursors and supporting cells were further analyzed. (a') SWNE plot of hair cells and supporting cells from OD and VS utricles, showing 8 distinct cell clusters following reclustering at resolution of 0.2. (b-b'') The *chooseR* algorithm was used to identify the optimal resolution for cell clustering in an unbiased manner. b shows the Silhouette distribution plot with x-axis indicating tested resolutions and y-axis showing the silhouette scores. Each dot represents a cluster for each of the tested resolutions on the x-axis. Medians with 95% confidence intervals are shown. Red vertical line shows best resolution and blue horizontal line shows the decision threshold. Based on this plot, a resolution of 0.2 was chosen. UMAP plot (b') with a clustering resolution of 0.2 shows silhouette scores with1 (red) indicating highest confidence in cluster assignment. Nearly all cells have high silhouette scores. b'' represents average co-clustering frequency at a 0.2 resolution following 100 random sub-samples of the data using 80% of the cells, indicating that the bootstrapping test produces 13 clusters consistently. This same method in b-b'' was used for re-clustering of cells in a'. (c) Decomposed SWNE plots showing 8 clusters (0-7) in both OD and VS utricles. (d) Five clusters of supporting cells, 1 of hair cell precursors, and 2 of hair cells were found. (e) Violin plot of mitochondrial gene percentages by cell cluster in d. (f) Dot plots showing the top 3 highly enriched genes within these 8 cell clusters, colored by relative expression from -1 (blue), 0 (purple), and 2 (red). The size of dots represents percent expressed. (g) Cryosections of VS utricle showing that *PPP1R27* or *LRCC10B* (red) was expressed in $MYO7A^+$ (green) hair cells and *CD9* (gray) is expressed in $MYO7A^-/GFAP^+$ (green/red) supporting cells. SWNE plots showing expression of *PPP1R27* or *LRCC10B* in hair cells and *CD9* in supporting cells. Scale bar=25 μm in g. Source data are provided as a Source Data file.

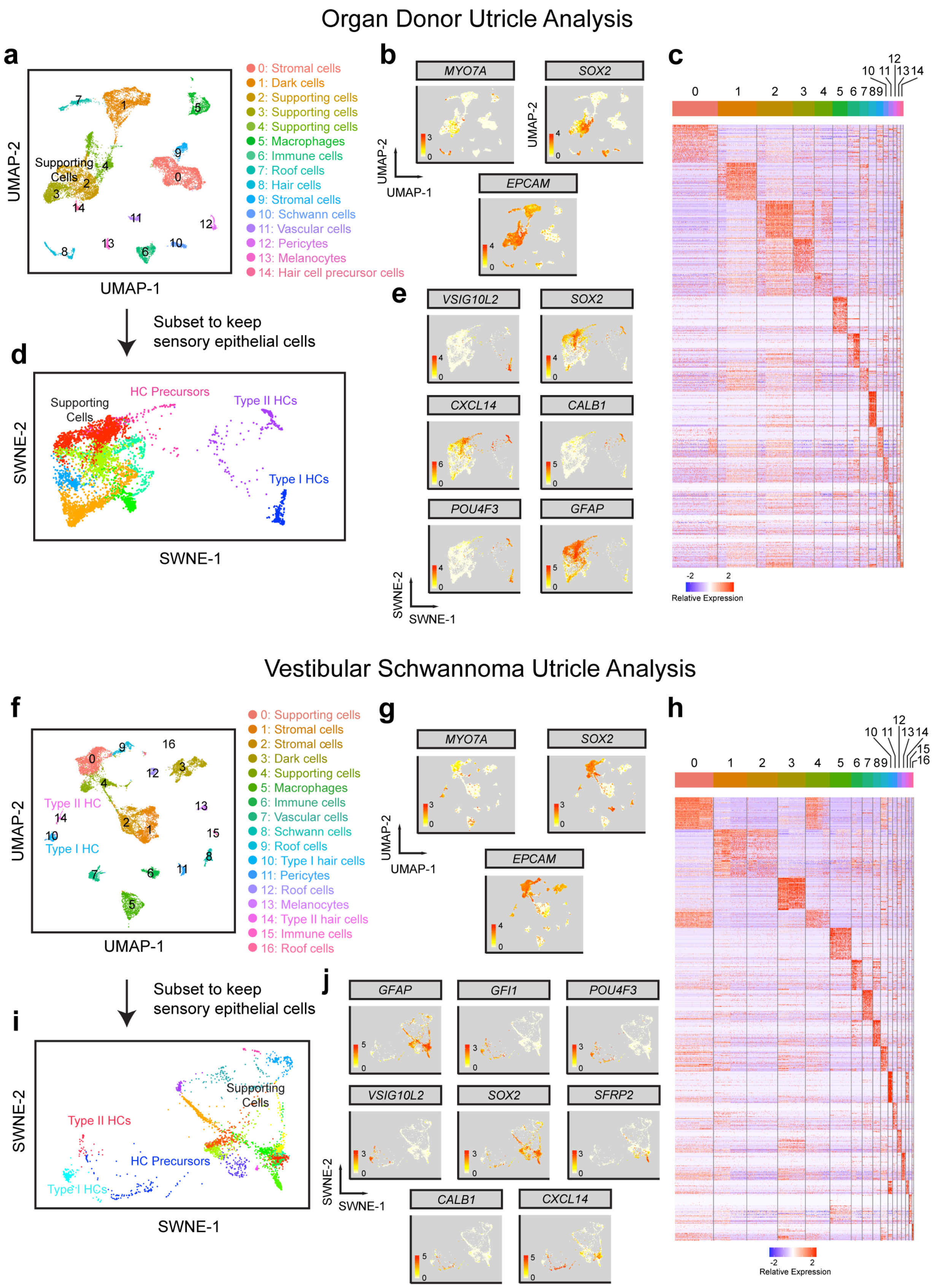
Organ Donor Utricle Analysis
a
UMAP-2
UMAP-1
Supporting Cells
0: Stromal cells
1: Dark cells
2: Supporting cells
3: Supporting cells
4: Supporting cells
5: Macrophages
6: Immune cells
7: Roof cells
8: Hair cells
9: Stromal cells
10: Schwann cells
11: Vascular cells
12: Pericytes
13: Melanocytes
14: Hair cell precursor cells
b
MYO7A
SOX2
EPCAM
c
Relative Expression
d
Subset to keep sensory epithelial cells
SWNE-2
SWNE-1
Supporting Cells
HC Precursors
Type II HCs
Type I HCs
e
VSIG10L2
SOX2
CXCL14
CALB1
POU4F3
GFAP
Vestibular Schwannoma Utricle Analysis
f
Type II HC
Type I HC
0: Supporting cells
1: Stromal cells
2: Stromal cells
3: Dark cells
4: Supporting cells
5: Macrophages
6: Immune cells
7: Vascular cells
8: Schwann cells
9: Roof cells
10: Type I hair cells
11: Pericytes
12: Roof cells
13: Melanocytes
14: Type II hair cells
15: Immune cells
16: Roof cells
g
MYO7A
SOX2
EPCAM
h
Relative Expression
i
Subset to keep sensory epithelial cells
Supporting Cells
Type II HCs
HC Precursors
Type I HCs
j
GFAP
GFI1
POU4F3
VSIG10L2
SOX2
SFRP2
CALB1
CXCL14

Supplementary Figure 3. Sensory and non-sensory cell types in utricles from vestibular schwannoma and organ donor patients.

(a) UMAP plot of organ donor utricle cells. Fifteen cell clusters were identified, and marker genes were used to annotate the sensory and non-sensory cell types. (b) Expression of established markers of epithelial cells (*EPCAM*), non-sensory cells and type II hair cells (*SOX2*) and sensory cells (*MYO7A*) within the cell clusters. (c) Heatmap showing differentially expressed genes among the 15 cell clusters. A full list of these genes is found in the Source Data file. (d) SWNE plot of hair cell subtypes, putative hair cell precursor cells, and supporting cells from organ donor utricles following cell subsetting. (e) SWNE plots displaying enrichment of *SOX2*, *GFAP*, and *CXCL14* in supporting cells, *POU4F3* in both type I and II hair cells, *CALB1* in type II hair cells, and *VSIG10L2* in type I hair cells. (f) UMAP plot of vestibular schwannoma utricle cells. Seventeen cell clusters were identified, and marker genes were used to annotate the sensory and non-sensory cell types. (g) Expression of established markers of epithelial cells (*EPCAM*), non-sensory cells and type II hair cells (*SOX2*) and sensory cells (*MYO7A*) within the cell clusters. (h) Heatmap showing differentially expressed genes among the 17 cell clusters. A full list of these genes is found in the Source Data file. (i) SWNE plot of hair cell subtypes, putative hair cell precursor cells, and supporting cells from vestibular schwannoma utricles following cell subsetting. (j) SWNE plots displaying enrichment of *SOX2*, *GFAP*, *SFRP2*, *CXCL14* in supporting cells, *POU4F3* and *GFI1* in both type I and II hair cells, *CALB1* in type II hair cells, and *VSIG10L2* mostly in type I hair cells. Source data are provided as a Source Data file.

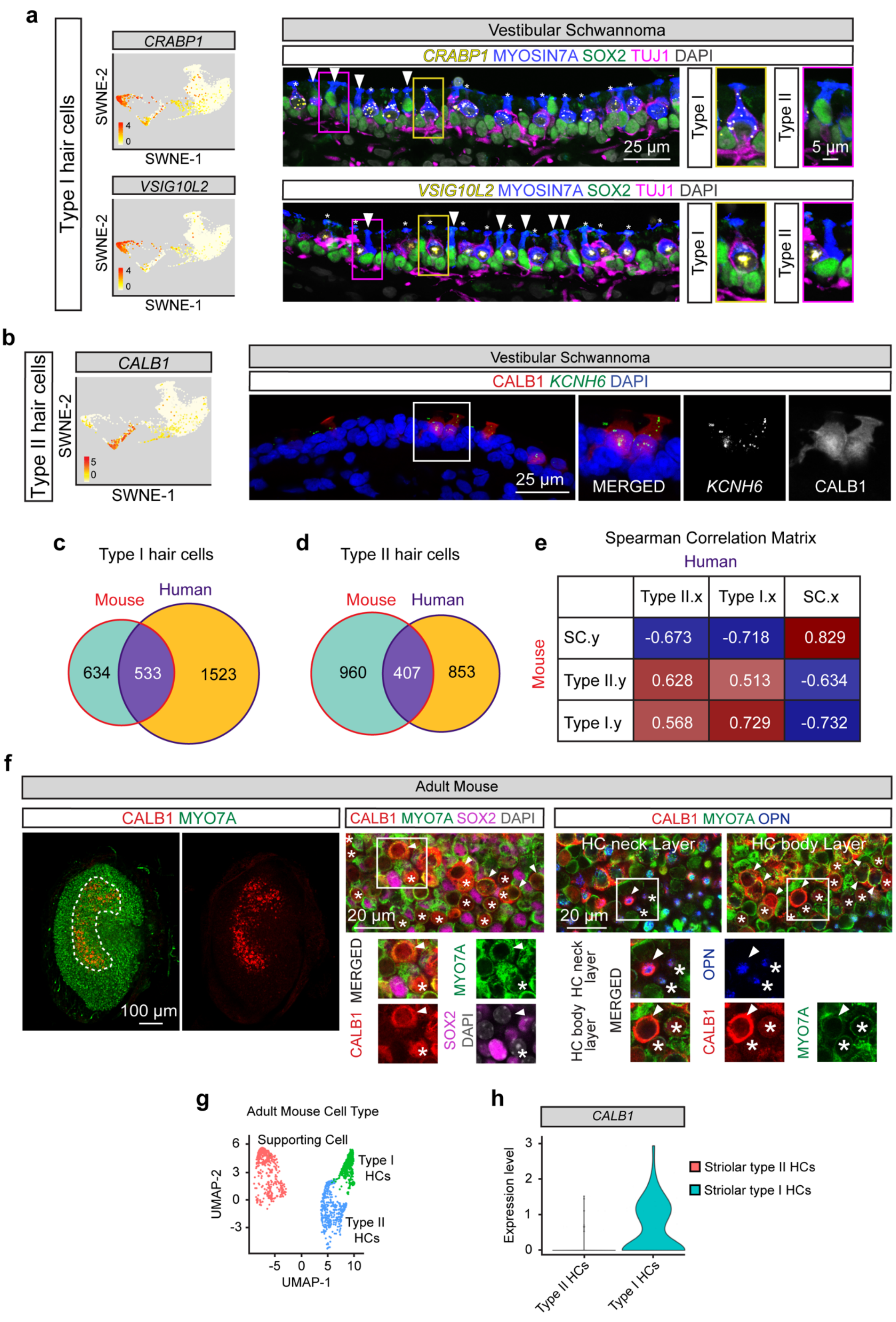
a
Type I hair cells
CRABP1
VSIG10L2
SWNE-2
SWNE-1
Vestibular Schwannoma
CRABP1 MYOSIN7A SOX2 TUJ1 DAPI
VSIG10L2 MYOSIN7A SOX2 TUJ1 DAPI
Type I
Type II
25 µm
5 µm
b
Type II hair cells
CALB1
Vestibular Schwannoma
CALB1 KCNH6 DAPI
25 µm
MERGED
KCNH6
CALB1
c
Type I hair cells
Mouse
Human
634
533
1523
d
Type II hair cells
Mouse
Human
960
407
853
e
Spearman Correlation Matrix
Human
Mouse
Type II.x
Type I.x
SC.x
SC.y -0.673 -0.718 0.829
Type II.y 0.628 0.513 -0.634
Type I.y 0.568 0.729 -0.732
f
Adult Mouse
CALB1 MYO7A
CALB1 MYO7A SOX2 DAPI
CALB1 MYO7A OPN
HC neck Layer
HC body Layer
100 µm
20 µm
MERGED
MYO7A
CALB1
SOX2 DAPI
HC neck layer
HC body layer
OPN
g
Adult Mouse Cell Type
Supporting Cell
Type I HCs
Type II HCs
UMAP-1
UMAP-2
h
CALB1
Expression level
Striolar type II HCs
Striolar type I HCs
Type II HCs
Type I HCs

Supplementary Figure 4. Genes enriched in hair cell and species comparison.

(a) SWNE plots showing enriched expression of *CRABP1* and *VSIG10L2* expression in type I hair cells. Cryosection of vestibular schwannoma samples immunolabeled for MYO7A (blue), SOX2 (green), TUJ1 (magenta) and DAPI (gray) combined with *in situ* hybridization showing expression of *CRABP1* or *VSIG10L2* mRNA (yellow) in type I hair cells (asterisks). Type II hair cells marked with arrowheads. (b) SWNE plot showing enriched expression of *CALB1* in type II hair cells. Anti-CALB1 immunolabeling combined with *KCNH6 in situ* hybridization shows co-localization of these two type II hair cell markers in vestibular schwannoma samples. (c-d) Species comparison analysis of mouse and human type I and type II hair cell transcriptomes. Venn diagram of differentially expressed genes expressed in both human and mouse using method in Tosches et al., 2018. See the Source Data file for accompanying gene list. (e) Spearman correlation matrix of mouse and human type I, type II hair cell, and supporting cell transcriptomes. All comparisons show a statistically significant correlation, albeit moderate correlation of hair cell subtypes at 0.628 for type II hair cells and 0.729 for type I hair cells. There is robust correlation of supporting cells at 0.829. (f) Adult mouse utricle stained for CALB1 (red), MYO7A (green), SOX2 (magenta) and OPN (blue). CALB1 is expressed in the striolar region. High magnification images showing CALB1 expression in SOX2-negative OPN-positive striolar type I hair cell cytoplasm (arrowheads) and nuclei (asterisks). (g) UMAP plot showing type I, type II and supporting cells from adult mouse utricle single cell RNAseq data. (h) Violin plot showing enrichment of *CALB1* in *OCM*-positive striolar type I hair cells in mice. Scale bar=25 and 5 μm in a, 25 μm in b, 100 and 20 μm in f. Source data are provided as a Source Data file.

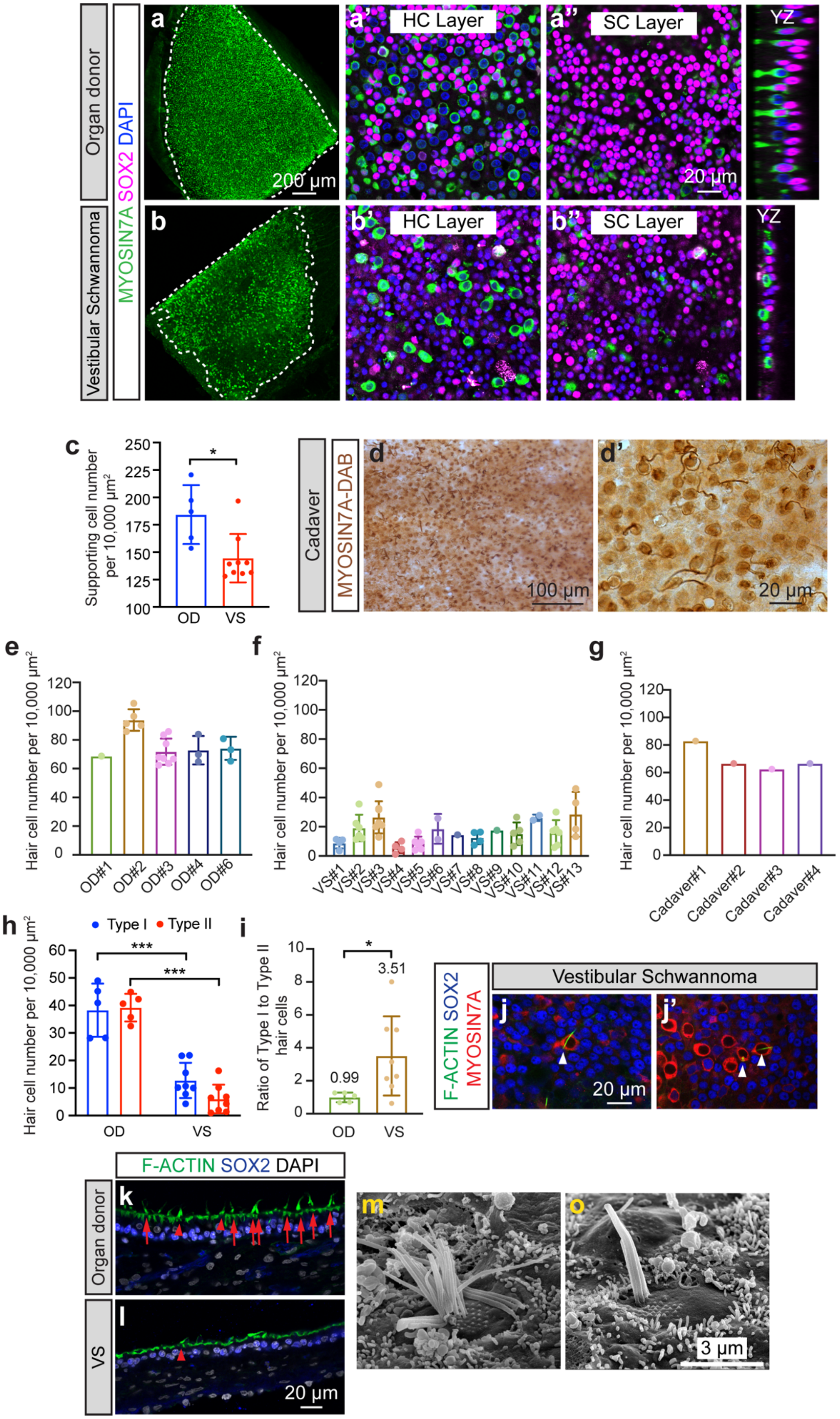

Organ donor
Vestibular Schwannoma
MYOSIN7A SOX2 DAPI
a
a'
HC Layer
a''
SC Layer
YZ
200 μm
20 μm
b
b'
b''
c
Supporting cell number per 10,000 μm²
OD
VS
Cadaver
MYOSIN7A-DAB
d
d'
100 μm
e
f
g
Hair cell number per 10,000 μm²
OD#1
OD#2
OD#3
OD#4
OD#6
VS#1
VS#2
VS#3
VS#4
VS#5
VS#6
VS#7
VS#8
VS#9
VS#10
VS#11
VS#12
VS#13
Cadaver#1
Cadaver#2
Cadaver#3
Cadaver#4
h
Type I
Type II
i
Ratio of Type I to Type II hair cells
3.51
0.99
Vestibular Schwannoma
F-ACTIN SOX2 MYOSIN7A
j
j'
F-ACTIN SOX2 DAPI
k
l
m
o
3 μm

Supplementary Figure 5. Degenerating hair cells in utricles from vestibular schwannoma patients.

(a-b’’) Organ donor and VS tissues were stained for MYO7A (green), SOX2 (magenta) and DAPI (blue). Low magnification images show fewer hair cells present in the sensory epithelium (dashed lines). Representative high magnification images show loss of many hair cells in VS utricle, while most supporting cells remained. (c) Counts of supporting cells were significantly lower in VS tissues (n=9) compared to OD tissues (n=5). (d-d’) Additional examples of cadaveric utricle stained with MYO7A-DAB (brown) showing dense population of hair cells. (e-g) Quantification of hair cell number from utricles from individual ODs, VS patients and cadavers. Scatter plots represent individual areas sampled from each organ. (h) Quantification of type I and type II hair cells showing loss of both in VS (n=8) relative to OD (n=5) tissues. (i) The ratio of type I and type II hair cells was significantly higher in VS (n=8) than OD (n=5) tissues (j-j’) F-ACTIN (green) labeling actin-rich cables through the bodies of some hair cells (cytocauds, arrowhead) in VS tissues. (k-l) Images of sections of utricular sensory epithelium stained with F-ACTIN (green), SOX2 (blue) and DAPI (gray) in both OD and VS tissues. Many long bundles (arrow) and few short bundles (arrowhead) were found in OD tissue. Occasional short bundles (arrowhead) were present in VS tissue. (m-o) Scanning electron microscopy (SEM) of hair cells with damaged, disorganized hair bundle in VS tissues. Data shown as mean ± S.D. and compared using unpaired, Student’s t-tests and two-way ANOVA. *p=0.010 in c, ***p=0.0001 for type I and <0.001 for type II HCs in h, *p=0.0430 in i. n=5 for OD, 9 for VS tissues in c. n=5 for OD, 8 for VS tissues in h and i. Scale bar=200 and 20 μm in a and b, 100 and 20 μm in d-d’, 20 μm in j-l, 3 μm in m and o. Source data are provided as a Source Data file.

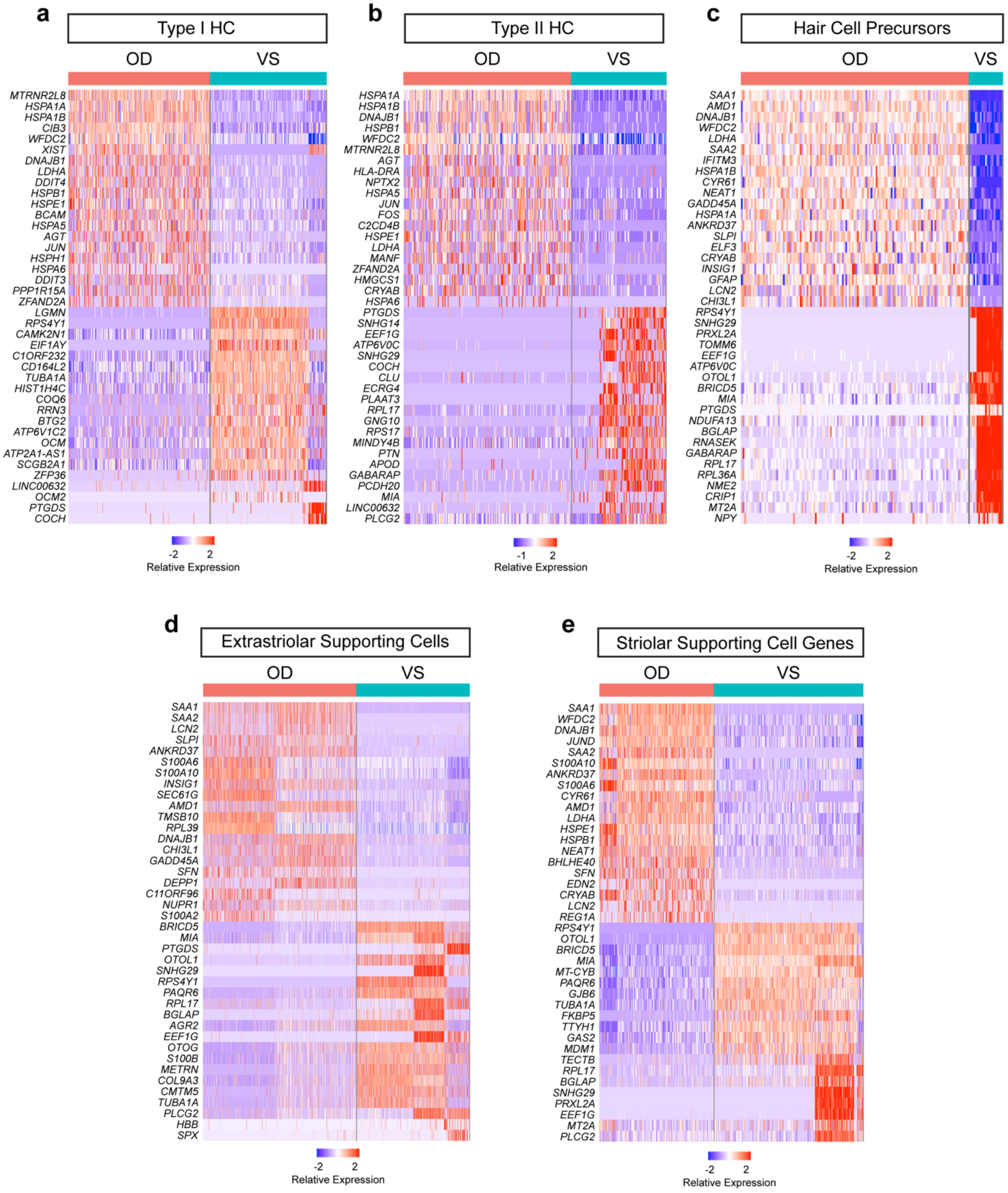
a
Type I HC
OD
VS
MTRNR2L8
HSPA1A
HSPA1B
CIB3
WFDC2
XIST
DNAJB1
LDHA
DDIT4
HSPB1
HSPE1
BCAM
HSPA5
AGT
JUN
HSPH1
HSPA6
DDIT3
PPP1R15A
ZFAND2A
LGMN
RPS4Y1
CAMK2N1
EIF1AY
C1ORF232
CD164L2
TUBA1A
HIST1H4C
COQ6
RRN3
BTG2
ATP6V1C2
OCM
ATP2A1-AS1
SCGB2A1
ZFP36
LINC00632
OCM2
PTGDS
COCH
-2
2
Relative Expression
b
Type II HC
OD
VS
HSPA1A
HSPA1B
DNAJB1
HSPB1
WFDC2
MTRNR2L8
AGT
HLA-DRA
NPTX2
HSPA5
JUN
FOS
C2CD4B
HSPE1
LDHA
MANF
ZFAND2A
HMGCS1
CRYAB
HSPA6
PTGDS
SNHG14
EEF1G
ATP6V0C
SNHG29
COCH
CLU
ECRG4
PLAAT3
RPL17
GNG10
RPS17
MINDY4B
PTN
APOD
GABARAP
PCDH20
MIA
LINC00632
PLCG2
-1
2
Relative Expression
c
Hair Cell Precursors
OD
VS
SAA1
AMD1
DNAJB1
WFDC2
LDHA
SAA2
IFITM3
HSPA1B
CYR61
NEAT1
GADD45A
HSPA1A
ANKRD37
SLPI
ELF3
CRYAB
INSIG1
GFAP
LCN2
CHI3L1
RPS4Y1
SNHG29
PRXL2A
TOMM6
EEF1G
ATP6V0C
OTOL1
BRICD5
MIA
PTGDS
NDUFA13
BGLAP
RNASEK
GABARAP
RPL17
RPL36A
NME2
CRIP1
MT2A
NPY
-2
2
Relative Expression
d
Extrastriolar Supporting Cells
OD
VS
SAA1
SAA2
LCN2
SLPI
ANKRD37
S100A6
S100A10
INSIG1
SEC61G
AMD1
TMSB10
RPL39
DNAJB1
CHI3L1
GADD45A
SFN
DEPP1
C11ORF96
NUPR1
S100A2
BRICD5
MIA
PTGDS
OTOL1
SNHG29
RPS4Y1
PAQR6
RPL17
BGLAP
AGR2
EEF1G
OTOG
S100B
METRN
COL9A3
CMTM5
TUBA1A
PLCG2
HBB
SPX
-2
2
Relative Expression
e
Striolar Supporting Cell Genes
OD
VS
SAA1
WFDC2
DNAJB1
JUND
SAA2
S100A10
ANKRD37
S100A6
CYR61
AMD1
LDHA
HSPE1
HSPB1
NEAT1
BHLHE40
SFN
EDN2
CRYAB
LCN2
REG1A
RPS4Y1
OTOL1
BRICD5
MIA
MT-CYB
PAQR6
GJB6
TUBA1A
FKBP5
TTYH1
GAS2
MDM1
TECTB
RPL17
BGLAP
SNHG29
PRXL2A
EEF1G
MT2A
PLCG2
-2
2
Relative Expression

Supplementary Figure 6. Comparative analysis of organ donor and vestibular schwannoma cell types.

(a-e) Heatmaps depicting integrated organ donor and vestibular schwannoma dataset analysis that systematically compares type I hair cells, type II hair cells, hair cell precursor, extrastriolar and striolar supporting cells between the two conditions. Source data are provided as a Source Data file.

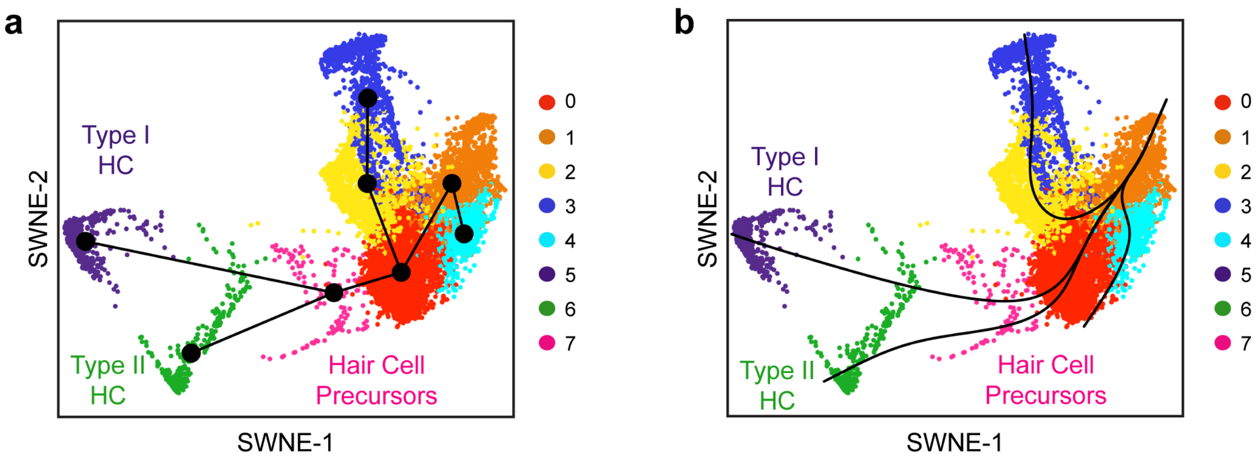

Supplementary Figure 7. *Slingshot* Trajectory Analysis.

(a) *Slingshot* was used to connect the cell clusters with cluster 1 supporting cells designated as the starting point and type I and type II hair cells designated as end points (clusters 5 and 6, respectively). (b) A total of 4 lineages were detected in this model with two lineages going toward type I and type II hair cells. Two other lineages are directed toward supporting cell subtypes.

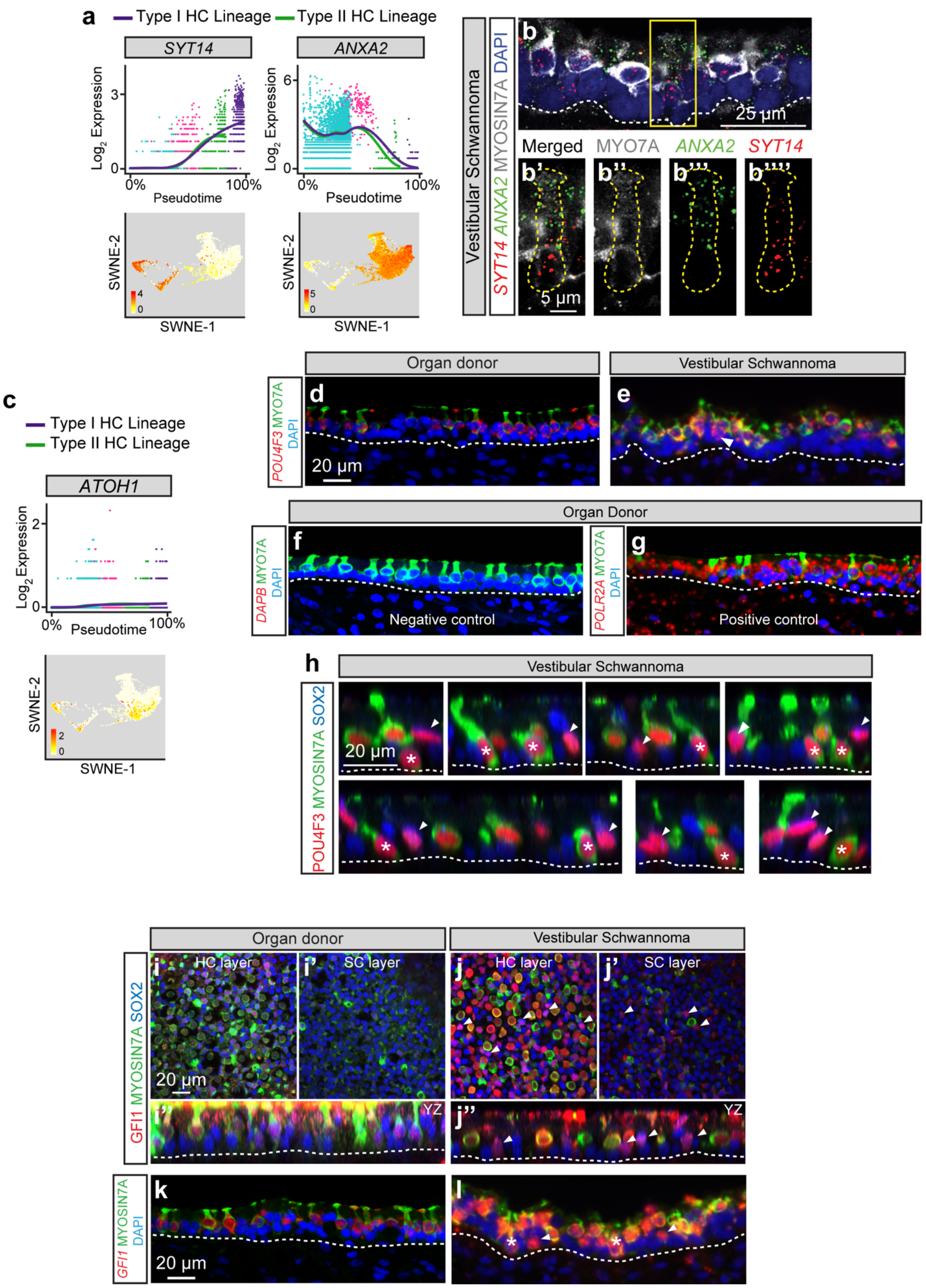

a
Type I HC Lineage
Type II HC Lineage
SYT14
ANXA2
Log2 Expression
Pseudotime
0%
100%
SWNE-1
SWNE-2
b
Vestibular Schwannoma
SYT14 ANXA2 MYOSIN7A DAPI
25 µm
Merged
MYO7A
ANXA2
SYT14
b'
b''
b'''
b''''
5 µm
c
ATOH1
d
e
Organ donor
Vestibular Schwannoma
POU4F3 MYO7A DAPI
20 µm
Organ Donor
f
g
DAPB MYO7A DAPI
POLR2A MYO7A DAPI
Negative control
Positive control
h
POU4F3 MYOSIN7A SOX2
i
i'
i''
j
j'
j''
HC layer
SC layer
YZ
GFI1 MYOSIN7A SOX2
k
l
GFI1 MYOSIN7A DAPI

Supplementary Figure 8. Characteristics of hair cell precursors in organ donor and vestibular schwannoma utricles.

(a) Pseudotime plots with generalized additive models depicting dynamic gene expressions along the supporting cell-to-type I hair cell and -type II hair cell lineages, with hair cell precursors colored in magenta. These plots predict that hair cell precursors co-express *SYT14*, *ANXA2*, and *MYO7A*. Both *SYT14* and *MYO7A* expression is upregulated while *ANXA2* expression is downregulated in both lineages. SWNE expression plots show the 2-dimensional locations of cells expressing these genes. (b-b'''') Cryosection of a VS utricle showing an elongated hair cell-precursor-like cell expressing *SYT14* (green), *ANXA2* (red), MYO7A (gray). White dashed line marks the basement membrane. Magnified images showing a *SYT14*/*ANXA2*/MYO7A-positive cell (yellow dashed line), whose nucleus is located close to the basement membrane below that of other hair cells. (c) A pseudotime plot showing *ATOH1* expression along the supporting cell-to-type I hair cell and -type II hair cell lineages. SWNE expression plot showing cells expressing *ATOH1*. (d-e) Cryosections of OD and VS tissues processed for *in situ* hybridization with probes directed against *POU4F3* (red) with immunostaining for MYO7A (green) and DAPI (blue). A *POU4F3*$^+$/MYO7A$^-$ hair cell precursor shown in VS tissue (arrowheads). (f-g) Negative (*DapB*) and positive (*POLR2A*) controls in VS tissues. (h) Representative examples of POU4F3$^+$/SOX2$^+$/ MYO7A$^+$ hair cell precursors (asterisks). These cells display nuclei at the supporting cell level, and below those of other hair cells in the VS tissue. The basal end of their cell bodies also abuts the basement membrane. (i-j'') More GFI1$^+$/SOX2$^+$/MYO7A$^-$ (red, blue, green) hair cell precursors (arrowhead) were present in VS tissue than OD tissues. Representative orthogonal view shows GFI1$^+$/SOX2$^+$/MYO7A$^-$ cells (arrowheads). (k-l) Many GFI1$^+$/MYO7A$^-$ hair cell precursors (red, green) were found in VS tissues (arrowhead). Some GFI1$^+$/SOX2$^+$/MYO7A$^+$ hair cell precursor cells (asterisks) were found in VS tissues with their nuclei similar to those of supporting cells and adjacent to the basement membrane (dashed lines). Scale bar=25 and 5 μm in b, 20 μm in d-l.

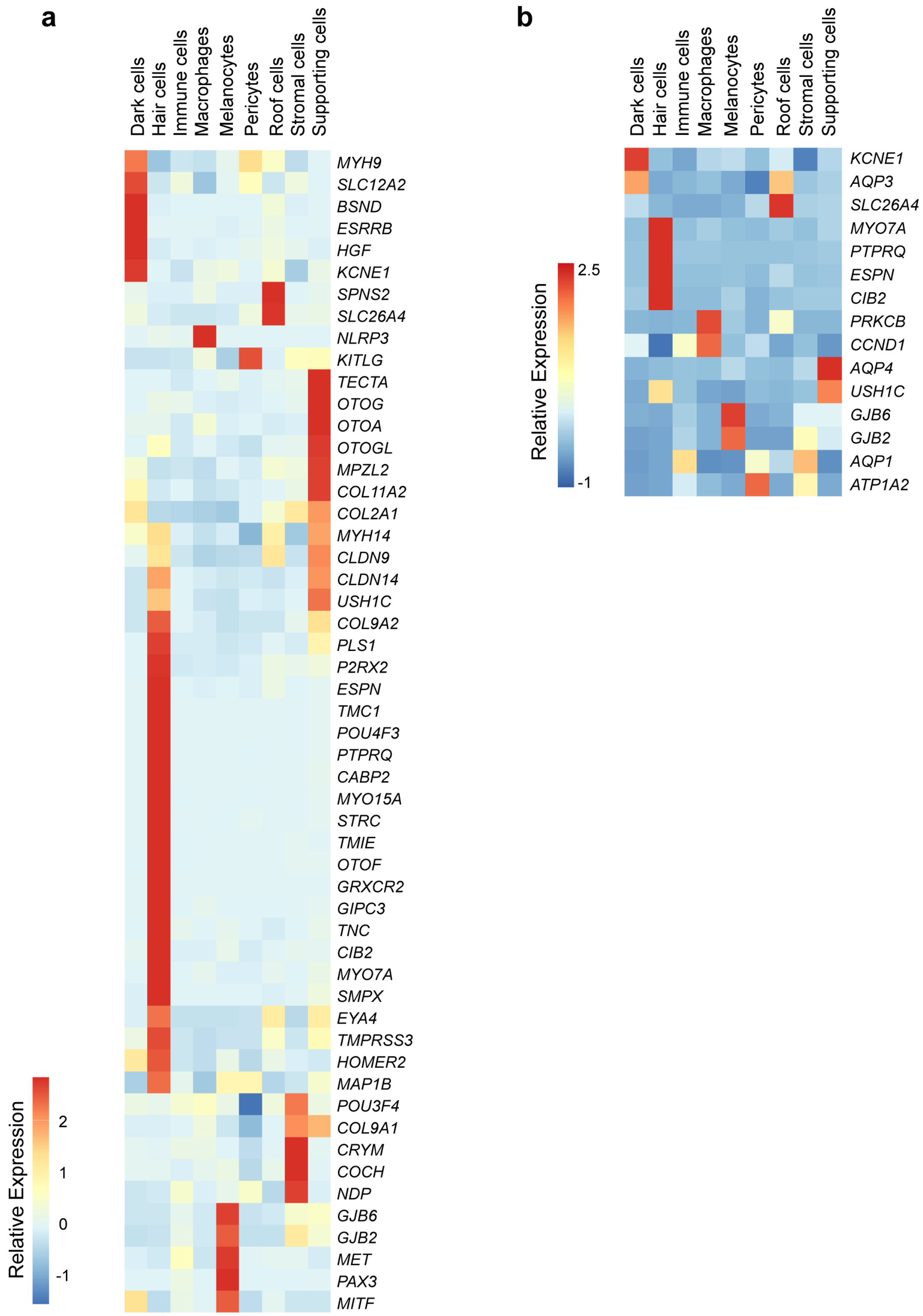
a
Dark cells
Hair cells
Immune cells
Macrophages
Melanocytes
Pericytes
Roof cells
Stromal cells
Supporting cells
MYH9
SLC12A2
BSND
ESRRB
HGF
KCNE1
SPNS2
SLC26A4
NLRP3
KITLG
TECTA
OTOG
OTOA
OTOGL
MPZL2
COL11A2
COL2A1
MYH14
CLDN9
CLDN14
USH1C
COL9A2
PLS1
P2RX2
ESPN
TMC1
POU4F3
PTPRQ
CABP2
MYO15A
STRC
TMIE
OTOF
GRXCR2
GIPC3
TNC
CIB2
MYO7A
SMPX
EYA4
TMPRSS3
HOMER2
MAP1B
POU3F4
COL9A1
CRYM
COCH
NDP
GJB6
GJB2
MET
PAX3
MITF
Relative Expression
2
1
0
-1
b
Dark cells
Hair cells
Immune cells
Macrophages
Melanocytes
Pericytes
Roof cells
Stromal cells
Supporting cells
KCNE1
AQP3
SLC26A4
MYO7A
PTPRQ
ESPN
CIB2
PRKCB
CCND1
AQP4
USH1C
GJB6
GJB2
AQP1
ATP1A2
Relative Expression
2.5
-1

Supplementary Figure 9. Expression patterns of genes associated with hearing loss and vestibular dysfunction.

(a-b) Heatmaps depicting relative expression of deafness related genes (a) and vestibular dysfunction related genes (b) averaged for each of the defined cell groups before any filtering. The groups of cells include dark cells, hair cells, immune cells, macrophages, melanocytes, roof cells, stromal cells, and supporting cells. This list of genes was adapted from the literature and from the Iowa Hereditary Hearing Loss webpage.

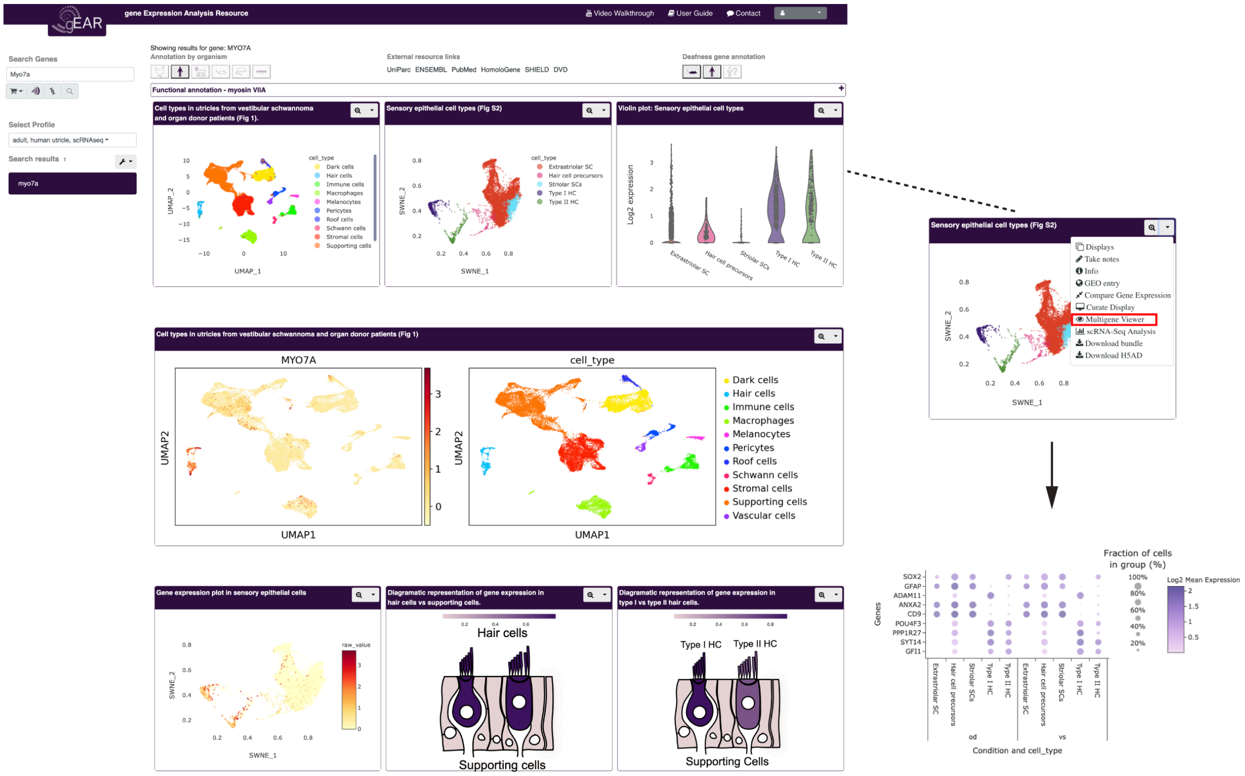

Supplementary Figure 10. gEAR profile demonstrating data accessibility.

This online platform allows users to easily access data to search genes of interest and to perform further independent analysis. Shown here is a view of the gEAR profile (https://umgear.org/p?l=human-utricle-sc-atlas) for this manuscript where the gene "Myo7a" is searched. Row one shows three panels that demonstrate a dynamic plot of the data, followed by subset one focusing on hair cells, supporting cells, and hair cell precursors, and finally a violin plot demonstrating expression values for each category of cells as indicated in the figure. Further analysis can be accessed by clicking the drop-down menu that includes Multigene Viewer as demonstrated on the right side. As an example, the genes validated in this manuscript are compared to each other and between conditions of vestibular schwannoma or organ donor. The second and third panels demonstrate expression patterns of the searched gene in both the entire dataset without subsetting, and the focused subset group of hair cells, supporting cells, and hair cell precursors. Finally, the last two panels of the third row have cartoon diagrams with gene expression levels superimposed with the middle panel focusing on hair cells versus supporting cells, and the third panel focusing on type I hair cells versus type II hair cells.
