# Supplementary material for: Single-cell transcriptomic atlas reveals increased regeneration in diseased human inner ear balance organs
Source: Nat Commun. 2024 Jun 6;15:4833. doi: 10.1038/s41467-024-48491-y (PMC11156867; doi:10.1038/s41467-024-48491-y)
Supplement: Supplementary file 3 — Description of Additional Supplementary Files [file 41467_2024_48491_MOESM3_ESM.pdf]

Description of Additional Supplementary Files

File name: Supplementary Data 1

Description: Patient demographic and medical information (related to all figures). Correlation between patient factors and utricle histology.
