# Supplementary material for: Single-cell transcriptomic atlas reveals increased regeneration in diseased human inner ear balance organs
Source: Nat Commun. 2024 Jun 6;15:4833. doi: 10.1038/s41467-024-48491-y (PMC11156867; doi:10.1038/s41467-024-48491-y)
Supplement: Supplementary file 5 — Reporting Summary [file 41467_2024_48491_MOESM5_ESM.pdf]

nature portfolio

Corresponding author(s): Taha Jan and Alan Cheng

Last updated by author(s): Mar 1, 2024

Reporting Summary

Nature Portfolio wishes to improve the reproducibility of the work that we publish. This form provides structure for consistency and transparency in reporting. For further information on Nature Portfolio policies, see our Editorial Policies and the Editorial Policy Checklist.

Statistics

For all statistical analyses, confirm that the following items are present in the figure legend, table legend, main text, or Methods section.

n/a	Confirmed	
☐	☒	The exact sample size (*n*) for each experimental group/condition, given as a discrete number and unit of measurement
☐	☒	A statement on whether measurements were taken from distinct samples or whether the same sample was measured repeatedly
☐	☒	The statistical test(s) used AND whether they are one- or two-sided<br>*Only common tests should be described solely by name; describe more complex techniques in the Methods section.*
☐	☒	A description of all covariates tested
☐	☒	A description of any assumptions or corrections, such as tests of normality and adjustment for multiple comparisons
☐	☒	A full description of the statistical parameters including central tendency (e.g. means) or other basic estimates (e.g. regression coefficient) AND variation (e.g. standard deviation) or associated estimates of uncertainty (e.g. confidence intervals)
☐	☒	For null hypothesis testing, the test statistic (e.g. *F*, *t*, *r*) with confidence intervals, effect sizes, degrees of freedom and *P* value noted<br>*Give P values as exact values whenever suitable.*
☐	☒	For Bayesian analysis, information on the choice of priors and Markov chain Monte Carlo settings
☐	☒	For hierarchical and complex designs, identification of the appropriate level for tests and full reporting of outcomes
☐	☒	Estimates of effect sizes (e.g. Cohen's *d*, Pearson's *r*), indicating how they were calculated

Our web collection on statistics for biologists contains articles on many of the points above.

Software and code

Policy information about availability of computer code

Data collection	Fiji V2.0.0, graphpad prism v9.3, adobe photoshop, R: A Language and Environment for Statistical Computing (4.0.4).
Data analysis	DoubletFinder (v2.0.3), scater (v1.18.6), Seurat (v4.0.2), tradeSeq (v1.5.07), slingshot (v1.8.0), pheatmap (v1.0.12), swne (v0.6.14), Ingenuity Pathway Analysis, DAVID.

For manuscripts utilizing custom algorithms or software that are central to the research but not yet described in published literature, software must be made available to editors and reviewers. We strongly encourage code deposition in a community repository (e.g. GitHub). See the Nature Portfolio guidelines for submitting code & software for further information.

Data

Policy information about availability of data

All manuscripts must include a data availability statement. This statement should provide the following information, where applicable:

- Accession codes, unique identifiers, or web links for publicly available datasets
- A description of any restrictions on data availability
- For clinical datasets or third party data, please ensure that the statement adheres to our policy

All data has been provided as main or supplementary figures or tables, and accessible via GEO and gEAR.

Research involving human participants, their data, or biological material

Policy information about studies with human participants or human data. See also policy information about sex, gender (identity/presentation), and sexual orientation and race, ethnicity and racism.

Reporting on sex and gender	Information on sex has been collected and data analyzed separately for sex. This has been described in the methods and results sections.
Reporting on race, ethnicity, or other socially relevant groupings	We did not collect information on race, ethnicity, or other socially relevant groupings.
Population characteristics	age 2-82
Recruitment	Patients were recruited via Stanford Hospital or the Donor Network West. This is described in details in the methods section. All vestibular schwannoma patients undergoing translabyrintine surgery were recruited. Organ donors were recruited by donor network West staff.
Ethics oversight	IRB approved at Stanford, Yale and UCLA.

Note that full information on the approval of the study protocol must also be provided in the manuscript.

Field-specific reporting

Please select the one below that is the best fit for your research. If you are not sure, read the appropriate sections before making your selection.

☒ Life sciences ☐ Behavioural & social sciences ☐ Ecological, evolutionary & environmental sciences

For a reference copy of the document with all sections, see nature.com/documents/nr-reporting-summary-flat.pdf

Life sciences study design

All studies must disclose on these points even when the disclosure is negative.

Sample size	Sample size was determined by available subjects.
Data exclusions	Only data from samples determined to have poor cell viability or poor RNA quality were excluded.
Replication	Data was replicated in triplicates or more.
Randomization	This was not relevant to our study.
Blinding	Data was blindly analyzed.

Reporting for specific materials, systems and methods

We require information from authors about some types of materials, experimental systems and methods used in many studies. Here, indicate whether each material, system or method listed is relevant to your study. If you are not sure if a list item applies to your research, read the appropriate section before selecting a response.

Materials & experimental systems

n/a	Involved in the study	
☐	☒	Antibodies
☒	☐	Eukaryotic cell lines
☒	☐	Palaeontology and archaeology
☐	☒	Animals and other organisms
☐	☒	Clinical data
☒	☐	Dual use research of concern
☒	☐	Plants

Methods

n/a	Involved in the study	
☒	☐	ChIP-seq
☒	☐	Flow cytometry
☒	☐	MRI-based neuroimaging

Antibodies

Antibodies used	MYOSIN7A Proteus Biosciences Cat Num 25-6790; RRID:AB_2314839<br>SOX2 Santa Cruz Biotechnology Cat Num sc-17320; RRID:AB_2286684<br>SOX2 R&D Systems Cat Num AF2018; RRID: AB_355110

TUJ1 Neuromics Cat Num MO15013; RRID:AB_2737114
CALBINDIN1 Cell Signaling Technologies Cat Num 13176; RRID:AB_2687400
GFI1 Gift from H. Bellen n/a
POU4F3 Santa Cruz Biotechnology Cat Num sc-81980; RRID:AB_2167543
GFAP Sigma Cat Num G3893; RRID:AB_477010
Osteopontin R&D Systems Cat Num AF808; RRID:AB_2194992
Phalloidin Thermo Fisher Scientific Cat Num A22287; RRID:AB_2620155
DAPI Thermo Fisher Scientific Cat Num D1306; RRID:AB_2629482
Alexa Fluor donkey anti-rabbit 488 Thermo Fisher Scientific Cat Num A21206; RRID:AB_2535792
Alexa Fluor donkey anti-rabbit 546 Thermo Fisher Scientific Cat Num A10040; RRID:AB_2534016
Alexa Fluor donkey anti-rabbit 647 Thermo Fisher Scientific Cat Num A31573; RRID:AB_2536183
Alexa Fluor donkey anti-goat 488 Thermo Fisher Scientific Cat Num A11055; RRID:AB_2534102
Alexa Fluor donkey anti-goat 546 Thermo Fisher Scientific Cat Num A11056; RRID:AB_142628
Alexa Fluor donkey anti-goat 647 Thermo Fisher Scientific Cat Num A21447; RRID:AB_141844
Alexa Fluor donkey anti-mouse 488 Thermo Fisher Scientific Cat Num A21202; RRID:AB_141607
Alexa Fluor donkey anti-mouse 546 Thermo Fisher Scientific Cat Num A10036; RRID:AB_2534012
Alexa Fluor donkey anti-mouse 647 Thermo Fisher Scientific Cat Num A31571; RRID:AB_162542
Alexa Fluor donkey anti-goat 405 Abcam Cat Num ab175664; RRID:AB_2313502
Alexa Fluor donkey anti-rabbit 594 Thermo Fisher Scientific Cat Num A-21207; RRID:AB_141637
Alexa Fluor donkey anti-goat 594 Thermo Fisher Scientific Cat Num A-11058; RRID. AB_2534105
Alexa Fluor donkey anti-mouset 594 Thermo Fisher Scientific Cat Num A-21203; RRID:AB_141633
Biotinylated antibodies Vector lab Cat Num BA-1000; RRID:AB_2313606

Validation

Describe the validation of each primary antibody for the species and application, noting any validation statements on the manufacturer's website, relevant citations, antibody profiles in online databases, or data provided in the manuscript.

Animals and other research organisms

Policy information about studies involving animals; ARRIVE guidelines recommended for reporting animal research, and Sex and Gender in Research

Laboratory animals	C57/bl6 mice
Wild animals	n/a
Reporting on sex	Data on sex was collected and described in Table S1.
Field-collected samples	n/a
Ethics oversight	IACUC approved at Stanford.

Note that full information on the approval of the study protocol must also be provided in the manuscript.

Clinical data

Policy information about clinical studies
All manuscripts should comply with the ICMJEguidelines for publication of clinical research and a completedCONSORT checklist must be included with all submissions.

Clinical trial registration	n/a
Study protocol	Stanford (IRB 27500, 48579, 38993, 50076), Donor network west (IRB #STAN-17-200), UCLA (IRB 10-001449), Yale (IRB 2000027777)
Data collection	2015-2022
Outcomes	na

Plants

Seed stocks	n/a
Novel plant genotypes	n/a
Authentication	n/a
